# Prevalence and distribution of odontogenic and nonodontogenic cysts in a Turkish Population

**DOI:** 10.4317/medoral.17088

**Published:** 2011-07-15

**Authors:** Aydan Açikgöz, Emel Uzun-Bulut, Bora Özden, Kaan Gündüz

**Affiliations:** 1 Professor, DDS, PhD, Department of Oral Diagnosis and Radiology, Ondokuz Mayıs University, School of Dentistry, Kurupelit- Samsun, Turkey; 2 Assistant Professor, DDS, PhD, Department of Oral and Maxillofacial Surgery, Ondokuz Mayıs University, School of Dentistry, Kurupelit- Samsun, Turkey; 3 Assistant Professor, DDS, PhD, Department of Oral Diagnosis and Radiology, Ondokuz Mayıs University, School of Dentistry, Kurupelit- Samsun, Turkey

## Abstract

Objective: To determine the relative frequency and distribution of odontogenic and nonodontogenic cysts in a large Turkish population. Study Design A retrospective survey of jaw cysts was undertaken at the Oral Diagnosis and Radiology and Oral and Maxillofacial Surgery Department, Ondokuz Mayıs University Dental School, Samsun, Turkey. Data were retrieved from clinical files, imaging, and histopathology reports from 2000 to 2008; a total of 12,350 patients were included. In each case, we analyzed age, gender, type and number of cysts, and cyst location. Imaging patterns and pathologies associated with cystic lesions were also determined. Results: The prevalence of odontogenic and nonodontogenic cysts was 3.51%; males were affected more frequently than females. There were 452 odontogenic cysts (98.5%) and seven nonodontogenic cysts (1.5%). The most frequent odontogenic cyst was radicular (54.7%), followed by dentigerous (26.6%), residual (13.7%), odontogenic keratocyst (3.3%), and lateral periodontal cyst (0.2%). Nasopalatine duct cyst (1.5%) was the only nonodontogenic cyst. By age, cysts peaked in the third decade (24.2%). Concerning location, no statistically significant difference was found between the maxilla and mandible (p>0.05). The most frequent radiological feature of these lesions was unilocular cyst (93.7%). Pathologies associated with cystic lesions occurred in 14.7%. Conclusion: The prevalence of both odontogenic and nonodontogenic cysts were lower than that reported in many other studies. In our study population, cysts were mainly inflammatory in origin.

** Key words:** Prevalence, odontogenic, nonodontogenic, cysts.

## Introduction

Odontogenic cysts (OCs) are one of the most common lesions affecting the jaws. OCs are derived from the epithelial component of the odontogenic apparatus or its remnants that lie entrapped within the bone or gingival tissue (1). Non-odontogenic cysts (nOC) also occur in the oral cavity; these cysts arise from ectoderm involved in facial tissue development (1). Odontogenic and non-odontogenic cysts are epithelial-lined cysts, with OCs classified as developmental or inflammatory and nOCs as developmental in origin (2-4). Demographic profiles of odontogenic and non-odontogenic cysts have been reported in various age groups in several European and non-European countries, including Spain (2), Brazil (3-7), Israel (8), Mexico (9,10), United Kingdom (11-13), France (14), Canada (15), Italy (16), Chile (17), Greece (18), Nigeria (19), Lithuania (20), and Thailand (21). These studies show variations in the distribution and frequency of cyst types. A review of the literature revealed that odontogenic cysts account for between 0.8% and 45.9% of the lesions diagnosed in the oral cavity (4,11); however, few reports can be found in the international literature on the prevalence of OC among the Turkish population (22). The purpose of this study was to evaluate the prevalence and distribution of these lesions in a large population over a period of nine years (2000-2008), and to compare results with findings in the literature.

## Material and Methods

 A retrospective survey was carried out among 12,350 individuals (5,500 males and 6,850 females, aged 6 to 90 years) who attended Ondokuz Mayıs University, Faculty of Dentistry, Department of Oral Diagnosis and Radiology and Oral and Maxillofacial Surgery in Samsun, Turkey, during the period 2000-2008 (9 years).

A total of 434 patients were diagnosed with cysts of the jaws based on histopathologic, clinical, and radiologic examination. Data were retrieved from patients’ clinical files, histopathology records, and imaging (panoramic and periapical radiographs in all cases, and CT in some cases). In every case the following information was obtained: age, gender, type and number of cysts, and lesion location. Patient’s age was reported as decade of life, from the first to the eighth decade or older. All radiographs were re-evaluated with regard to the localization, peripheral shape, and pathologies associated with cystic lesions. The maxilla was divided into three anatomic regions: anterior (from midline to distal surface of the canine), premolar (from mesial of the first premolar to distal of the second premolar), and molar (from mesial of the first molar and distally). The mandible was divided into 4 anatomic regions: anterior and premolar (both as described above), molar (from mesial of the first molar to distal of the third molar), and ascending ramus (upper portion of the ramus beyond the occlusal plane). The distal border of the lesion was accepted as the true localization.

**Figure 1:**
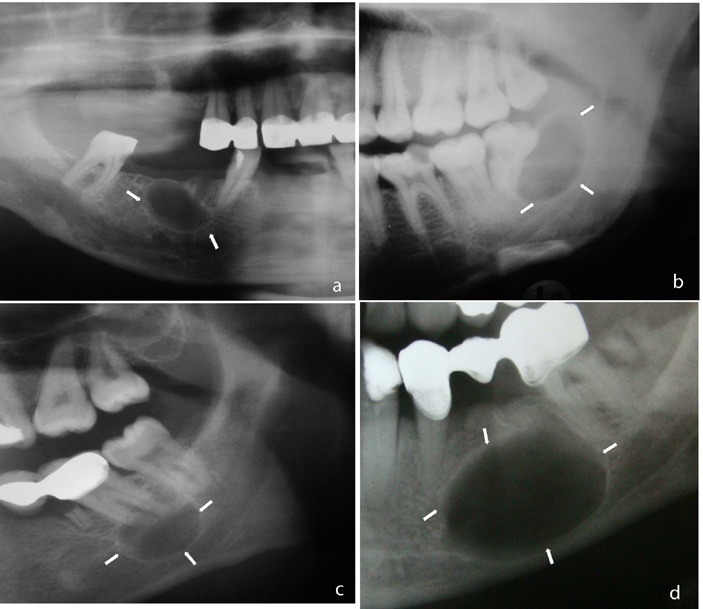
Unilocular cyst: well defined radiolucency with smooth border.

**Figure 2:**
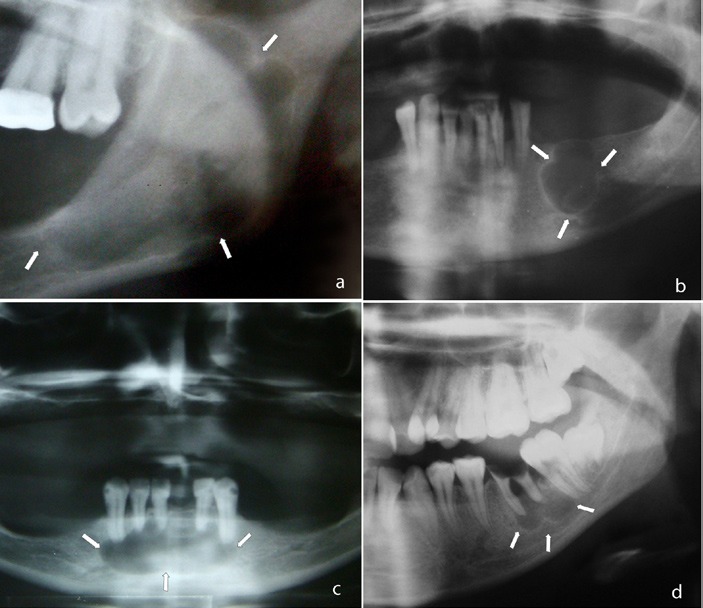
Lobulated cyst: unilocular lesion with scalloped border appearence.

**Figure 3:**
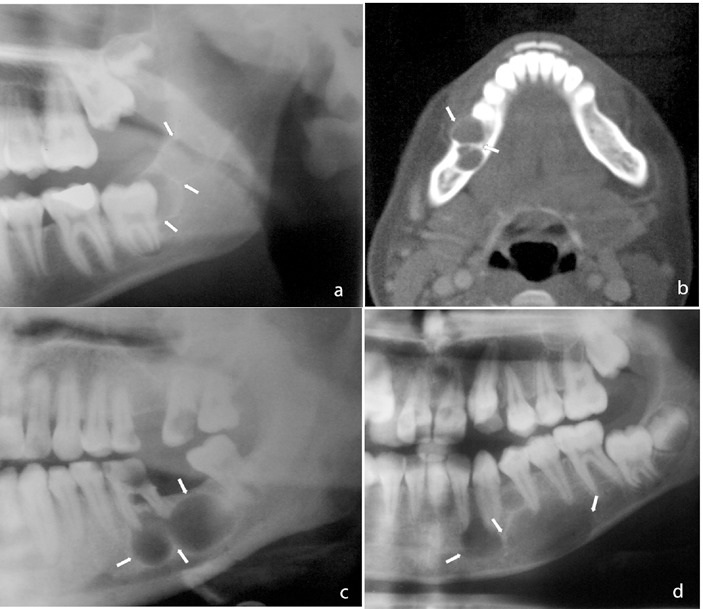
Multilocular cyst: cyst with internal structure that was predominantly radiolucent and divided into more than one cavity with thin septa (s).

The imaging patterns of peripheral cyst shape were classified as unilocular, lobulated, or multilocular. Well-defined radiolucency with smooth border was accepted as unilocular (Fig. 1), unilocular lesion with scalloped border appearance was considered as lobulated (Fig. 2), and lesions with internal structure that was predominantly radiolucent and divided into more than one cavity with thin septa(s) as mutilocular (Fig. 3).

Pathologies associated with cystic lesion were classified as follows: 1) displacement of implicated tooth, 2) displacement of adjacent tooth and/or root, 3) root resorption, and 4) preventing eruption of permanent teeth.

Z-test was used to determine differences in cyst numbers between males and females and to determine differences in cyst location in males and females. Chi-square tests were used to assess the statistical significance of differences between gender groups in the frequency distribution of categorical variables. All computational work was performed using Minitab (Minitab V. 13.20, 2000).

## Results

 Of the 12,350 individuals admitted during the period of study, 434 (233 males and 201 females) had at least one cyst, for a prevalence of 3.51%. A total of 459 cysts were diagnosed. Of these, 247 were in males (53.8%) and 212 cases in females (46.2%) (p<0.05). Four hundred and thirteen patients (95.2%) had a solitary cyst, 18 patients (4.1%) had two cysts, two patients (0.5%) had three cysts, and one patient (0.2%) had four cysts. Only five different diagnostic categories of OCs and one nOC variant were identified in this study ( Table 1). There were 452 odontogenic cysts (98.5%) and 7 nonodontogenic cysts (1.5%). Inflammatory OCs comprised 314 cases (69.5%) and developmental OCs represented 138 (30.5%). The most frequent OC was radicular (54.7%), followed by dentigerous (26.6%), residual (13.7%), odontogenic keratocyst (OKC) (3.3%), and lateral periodontal cyst (0.2%). Only nasopalatine duct cyst (NPDC) was found as nOC in our study. Regarding cyst distribution according to type, no statistically significant difference was found between men and women (p>0.05). ( Table 1) shows the distribution of cyst types according to diagnosis and gender.

The population with OC and nOC included patients from the first to eighth decades of life. Three hundred seventyeight cases (82.4%) were found in the second, third, fourth, and fifth decades, with a peak in the third decade (111 cases, 24.2%). Most radicular cysts occurred in the fourth (25.9%) decade, dentigerous cysts in the third (34.4%) decade, residual cysts in the sixth 

( 25.4%) decade , and odontogenic keratocysts in the second (26.7%) and sixth (26.7%) decades. In the present study, a total of 51 patients (11.6%) were 6-16 years old. The female-to-male ratio was 1.1:1. A total of 52.9% of the odontogenic cysts were inflammatory (radicular cyst) and 47.1% were developmental (dentigerous cyst) in origin.

**Table 1:**
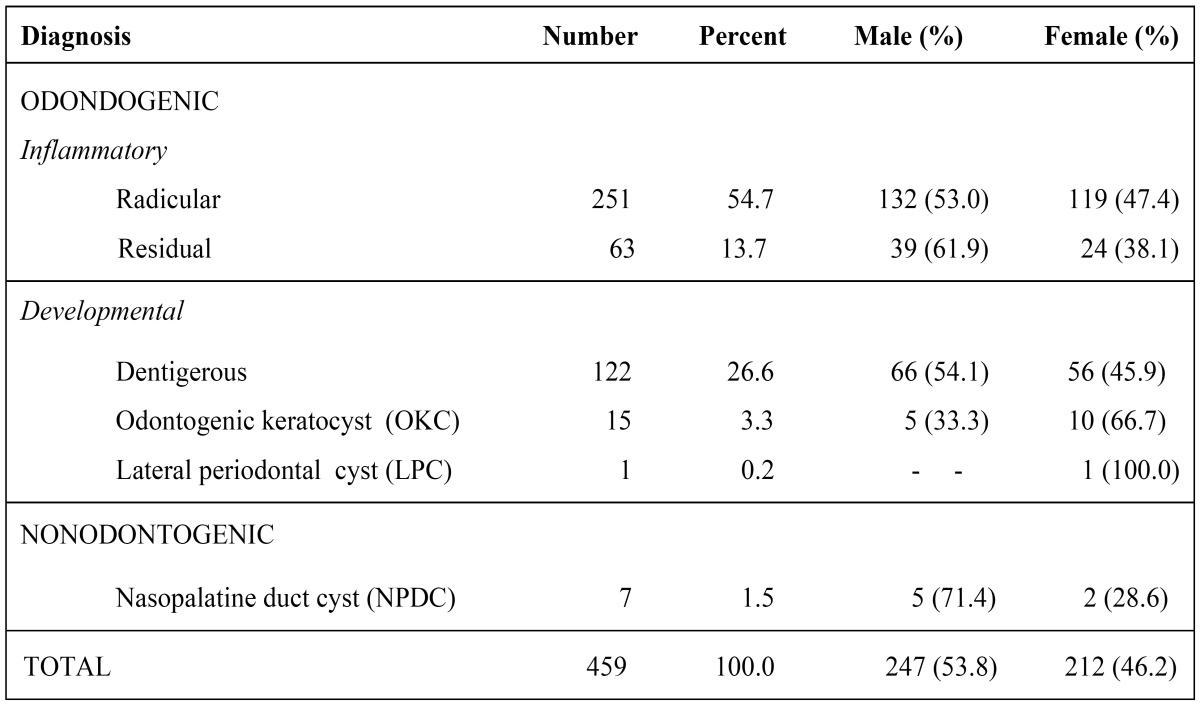
Distribution of cysts according to diagnosis and gender.

Regarding cyst distribution in the maxilla and mandible, no statistically significant difference was found between right and left sides (p>0.05). Nineteen cysts (4.1%), all localized to the anterior segment, were present on both right and left sides.

**Table 2:**
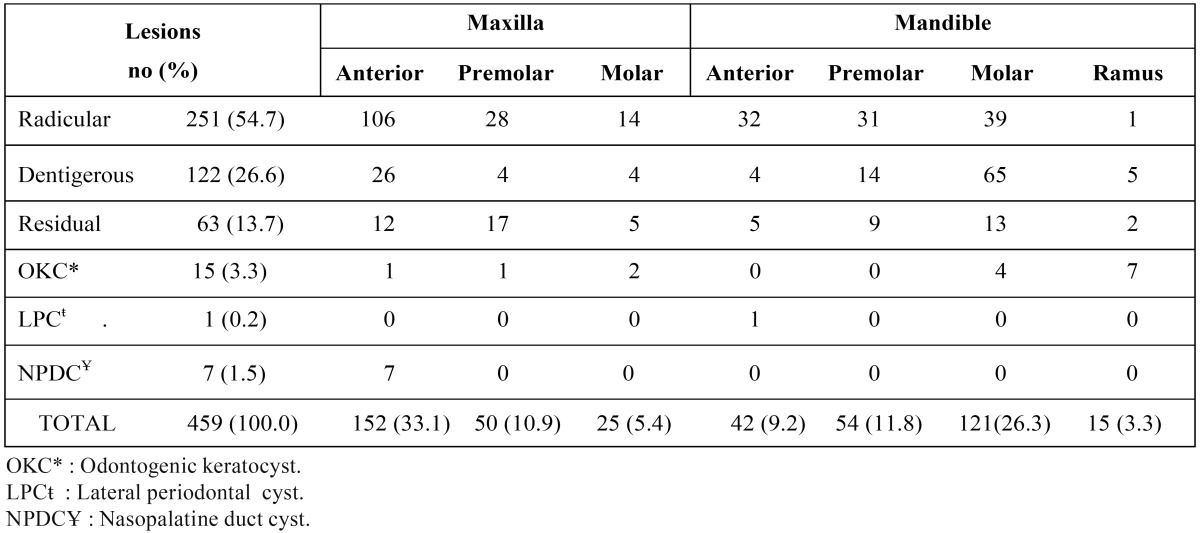
Distribution of 459 cysts according to anatomical site.

**Table 3:**
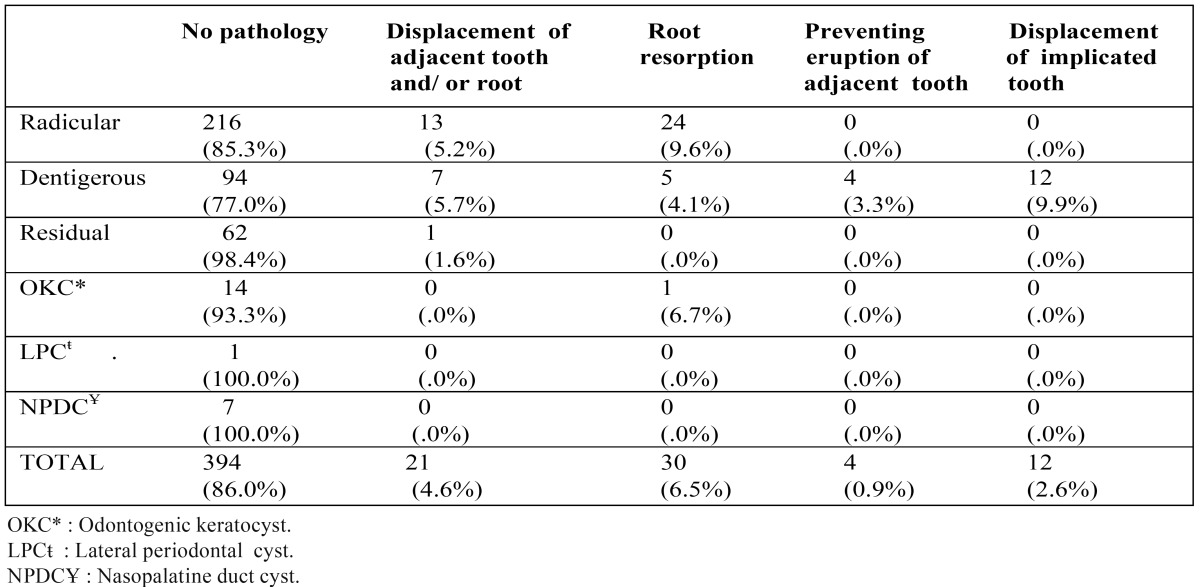
Pathologies associated with cystic lesion.

 Table 2 summarizes the localization of the cysts. The most frequent sites were the maxillary anterior region, which accounted for 152 cases (33.1%), followed by mandibular molar (121 cases, 26.4%), mandibular premolar (54 cases, 11.8%), and maxillary premolar (50 cases, 10.9%). The most frequent radiological feature of these lesions was unilocular (93.7%), followed by lobulated (5.0%) and multilocular (1.3%). Most lobulated and multilocular types of cysts were diagnosed as OKC.

 Table 3 reports pathologies associated with cystic lesions. Sixty-seven pathologies (14.6%) were found among 459 cases. Root resorption was the most common pathology (6.5%), followed by displacement of adjacent tooth and/or root (4.6%), displacement of implicated tooth (2.6%), and preventing eruption of adjacent tooth (0.9%). Radicular and dentigerous cysts were the most frequent types associated with pathologies. Root resorption was found more commonly in radicular cysts; displacement of implicated teeth and preventing eruption of adjacent teeth were seen most commonly with dentigerous cysts. The association of root resorption with radicular cysts was statistically significant (p<0.001) and the association of dentigerous cysts with preventing eruption of adjacent teeth was statistically significant (p<0.001).

## Discussion

 The present study is the largest series of cystic lesions of the jaws in a Turkish population described in the literature. Over a nine-year period, the prevalence of odontogenic and nonodontogenic cysts was 3.51%. Odontogenic cysts were diagnosed in 3.45% of all cases. This is lower than the range of 5.4%-33.8% reported from many European and non-European countries ( Table 4). Also, nOC in this study represented about 0.06% of all cases, whereas a higher rate was reported in most previous studies, including Brazil 0.5% (4), UK 1.5% and 1.3% (12,13), Canada 1.01% (15), and Sicily 2.82% (16). The variation in prevalence can be explained by differences in the study design. In most previously reported studies, in our opinion, the maxillofacial histopathologic biopsy slides were derived from pathology institutes and multicenter locations. Thus the total number of cases was probably increased in comparison with our study. In the present study we used only the cases presenting to our dental clinic. Another potential factor that influences the variation in results is the use of different cyst classification schemes. For example, in some studies non-epithelial-lined cysts and cysts of the soft tissues were also included (12,13,16,18,19,21).

**Table 4:**
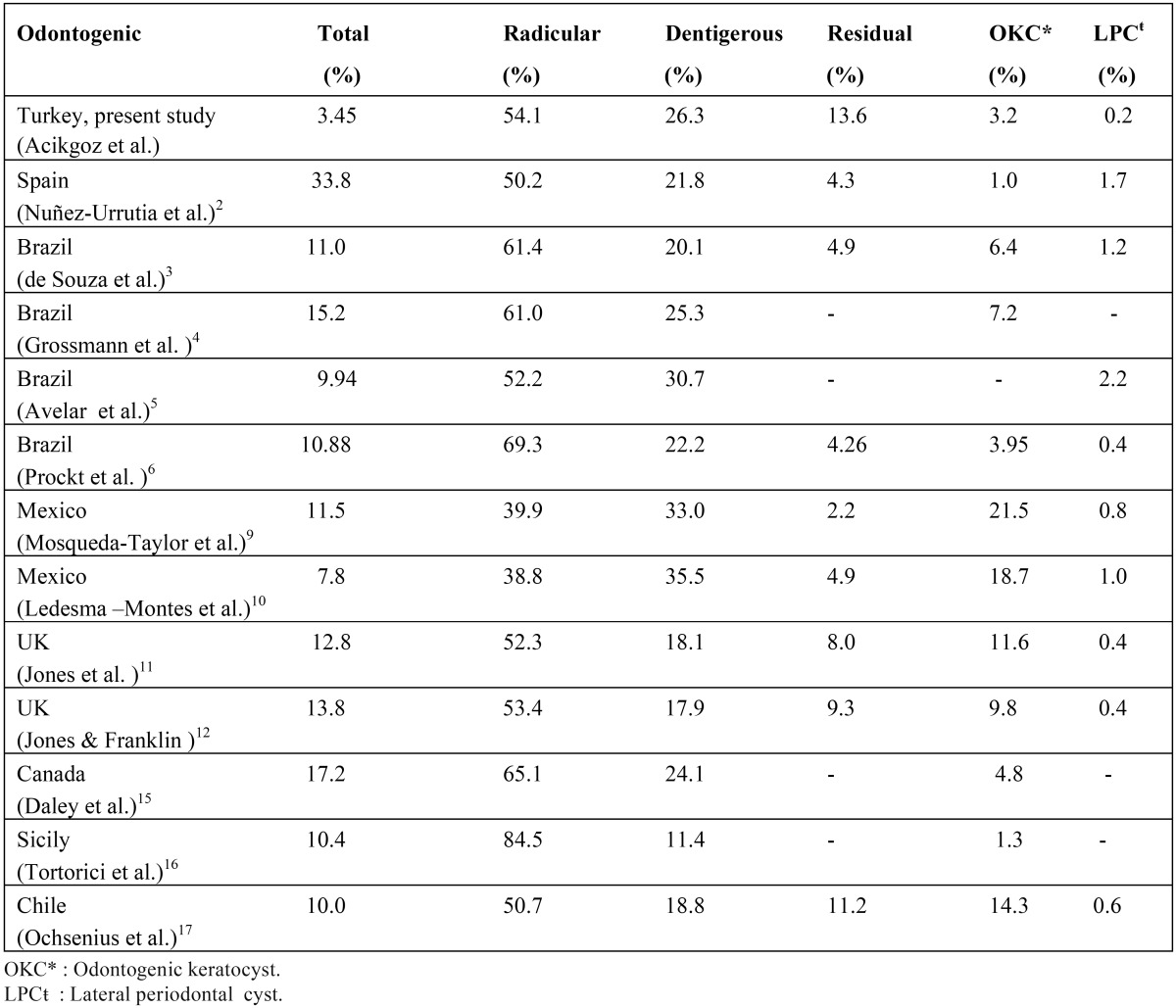
Geographic variation of odontogenic cysts from selected references.

In our study, cysts occurred in adult men more frequently than in women, which is similar to previous studies (2,5,2, 9-11, 13-17, 19,20,22). In some of these studies (2,5,9,14,16) it has been claimed that the greater prevalence in males is possibly explained by the fact that men customarily have worse oral hygiene habits and are more susceptible to trauma than women, both of these factors may lead to cyst formation. 

No statistically significant difference was found for location (maxilla versus mandible) in our study, in agreement with findings reported by Koseoglu et al. (22). However, many previous studies have reported that the maxilla was the anatomic site most often affected by odontogenic cysts (3,4,6, 16-20), whereas Nuñez-Urrutia et al. (2), Avelar et al. (5), and Meningaud et al. (14) reported that the mandible was the most frequently affected site. There is no definitive explanation in the literature related to site variations.

This study confirms that inflammatory cysts are the most frequently seen odontogenic cysts in the adult population, which is in agreement with most other reports (2-6, 11-17, 20). In our study, 52.9% of the odontogenic cysts were inflammatory and 47.1% were developmental in origin among children (ages 6-16), which is in agreement with a study conducted by Jones and Franklin (12) in the United Kingdom. However, most studies report a greater number of developmental cysts in children, such as those reported in Brazil (3,7), Israel (8), Greece (18), and Thailand (21). The reason for this difference may be explained by differences in the age range studied, the prevalence of caries, and oral health status. In our country, the rate of caries is high in the 5-12 year old age group. Prockt et al. (6) and Mosqueda-Taylor et al. (9) reported that an individual’s socioeconomic situation may also be an important factor. 

Radicular cysts with a predilection for the maxilla were the most frequent OC in this study. This confirms the findings of other studies, with variations only in percentages ( Table 4). Radicular cysts develop after pulp necrosis. These cysts remain and continue to develop if surgery is not performed. The frequency of dental visits and rate of dental surgery, in addition to factors affecting dental clinic attendance (fear, economic condition) result in the avoidance of necessary treatments. Overall, the incidence of caries in different population may explain this difference in cyst occurrence. The most common site involved was the maxillary anterior region in this study. This is in agreement with other studies (2,6,10,11,16,17,22). The reason for this could be related to the extra care provided to anterior teeth for aesthetic purposes, although the extraction ratio is higher in the posterior teeth group.

Dentigerous cysts are defined by Slootweg (1) as those surrounding the crown of a tooth that has not migrated into the oral cavity, but still lies buried in the jaw bone. Our finding that dentigerous cyst is the second most frequent odontogenic cyst after radicular cyst is in agreement with most other reports ( Table 4). However, in their Turkish study from Istanbul, Koseoglu et al. (22) reported that OKC was the second most frequent cyst type, but their case series included only 90 patients whereas they evaluated only the group they had made a surgical intervention between 1998 and 2000. Dentigerous cysts showed a predilection for the posterior mandible followed by the anterior maxilla with a peak incidence in the second and third decades. This was expected because the lower third molars and upper canines are the most commonly impacted teeth. As dentigerous cysts arise from the dental follicle, they are more commonly associated with impacted teeth than any other cysts.

Residual cysts are retained radicular cysts from teeth that have been extracted (2,6,17). In the current study, residual cysts with predilection for the maxilla were the third most common OC. Residual cysts occurred in relatively older age groups, which is in agreement with previous studies (3,6,10,11,16,17,22). This situation may be explained by the fact that cystic lesions cause no clinical symptoms after tooth extraction and are detected several years later as an incidental radiographic finding. 

Odontogenic keratocysts were the fourth most common type of OC, constituting 3.3% of all OCs in the present study. A review of the literature revealed that OKCs account for 1.0% to 21.5% of the lesions diagnosed in the oral cavity ( Table 4), in agreement with our results. Although many authors report a higher incidence of these cysts in men than women (3,4, 2-11, 13,14,16,17,19), more females were affected than males in our study population. This is consistent with results reported by Nuñez-Urrutia et al. (2) for a Spanish population, Chirapathomsakul et al. (23) for a Thai population, and Koseoglu et al. (22) for a Turkish population. These results are thought to be more related to the type of populations studied than to specific characteristics of these cysts. Most previous studies have reported that about two-thirds of OKCs are found in the mandible, with 50% in the third molar-ascending ramus area (3,4,10,11,16,23). The localization of OKCs in this study was also quite similar to these reports, with 73% of lesions found in the mandible, and all lesions in this bone were localized in the posterior region. Gorlin-Goltz syndrome was found clinically in only one female patient with OKC; this patient had three cysts at the time of diagnosis. It was reported that in a patient with more than one OKC, clinicians should be aware of the probability of the syndrome (4). Because these cysts have a higher rate of recurrence, it is important to differentiate them from other cysts or intraosseous lesions of the jaw. However, it should be remembered that nowadays this lesion is considered as an odontogenic tumor according to the W.H.O. and renamed as keratocystic odontogenic tumors (KOT) (2,6,11,13). Nasopalatine duct cyst is the most common of the nOC and it is thought to derive from embryonic epithelial rests in the nasopalatine canal. A review of the literature showed that the prevalence of NPDC is between 1.3% and 2.2% (4,10,11), similar to our data (1.5%). 

In our study, radicular, residual, and dentigerous cysts were usually unilocular in shape with a distinct sclerosing margin on radiographic studies, in agreement with general opinion (24). Multilocular lesions with scalloped margins were mostly seen in OKCs and these appearance seems to be more characteristic for OKC. Yoshiura et al.(24) reported that a multilocular pattern was more frequent in the keratinized OKCs and keratinization is strongly related to cyst morphology. Unfortunately, as we were unable to compare histopathologic appearance with radiological findings, we cannot comment on these theories. In most cases, plain film radiography (PFR) was an adequate imaging modality (14) and was used to evaluate the peripheral shape of the cysts. However, it was reported that radiologic features of cysts can be confused in conventional radiology and that CT images should be used for classification of these lesions. CT is shown to be superior to PFR in demonstrating the distinct borders, real extensions, and relation with adjacent anatomical structures of cystic lesions. However, despite the advantages, CT should not be used routinely, and we performed this imaging only for large lesions and those suspicious of being a tumor.

Pathologies associated with cystic lesions were seen in 14.6% of the cases. Root resorption was mostly seen with radicular cysts and prevention of adjacent tooth eruption with dentigerous cysts. Only one pathology was found related to OKC, which was root resorption of an adjacent tooth. Similar to our findings, Chirapathomsakul et al. (23) reported only one root resorption with OKCs, and they suggested that root resorption was not a characteristic feature of OKCs.

In conclusion, this study presents a series of OC and nOC in a Turkish population, where the prevalence of jaw cysts (3.55%) was lower than that reported in many other studies worldwide. Among cystic lesions of the jaws in adult and child populations, most are inflammatory in origin. Most OC were radicular, followed by dentigerous and residual. Odontogenic keratocysts were found in 3.3%, and 6.7% of these lesions were related to Gorlin-Goltz syndrome. Sixty-eight pathologies (14.7%) associated with cystic lesions were found in 459 cysts in this series. A dental team should be aware of the incidence of odontogenic cysts and their clinicopathologic features, including most common location and age distribution. This knowledge would allow for early and accurate diagnosis and treatment of these lesions.
